# Multimodal closed-loop strategies for gait recovery after spinal cord injury and stroke via the integration of robotics and neuromodulation

**DOI:** 10.3389/fnins.2025.1569148

**Published:** 2025-07-17

**Authors:** Valeria de Seta, Simone Romeni

**Affiliations:** ^1^CHUV, Department of Clinical Neurosciences, University Hospital Lausanne, Lausanne, Switzerland; ^2^Bertarelli Foundation Chair in Translational Neural Engineering, Neuro-X Institute, Ecole Polytechnique Federale de Lausanne, Lausanne, Switzerland; ^3^Modular Implantable Neurotechnologies (MINE) Laboratory, Università Vita Salute San Raffaele & Scuola Superiore Sant'Anna, Milan, Italy

**Keywords:** gait recovery, robotic assistance, neuromodulation, multimodal approach, closed-loop technologies, MoBI, neuromotor disorders, personalized therapy

## Abstract

Restoring the ability to walk is a priority for individuals with neurological disorders or neurotraumatic injuries, given its significant impact on independence and quality of life. Multimodal closed-loop strategies that integrate robotic assistance and neuromodulation present promising avenues for personalized and physiological gait recovery. These approaches capitalize on residual motor activity, fostering neuroplasticity and motor relearning. This narrative review emphasizes the importance of mobile brain/body imaging (MoBI) for guiding the development of closed-loop systems that integrate volitional brain signals with residual motor activity in stroke and spinal cord injury patients. We explore the potential of rehabilitative and assistive interventional strategies based on robotic devices, such as exoskeletons and powered orthoses, and neuromodulation techniques like functional electrical stimulation and spinal cord stimulation. We highlight the limitations of the single interventional strategies and the potential of the synergistic combination of MoBI, robotics, and neuromodulation for gait recovery. By leveraging residual motor functions and integrating multimodal data from the different domains involved in motor recovery (i.e., brain, muscle, and biomechanics), the complementarity of these interventional strategies has the potential to enable dynamic patient-specific interventions. We outline a perspective framework on how future directions can exploit such integration to promote physiological recovery of lower limb functions and personalized therapies that are both challenging and feasible. Advancing along this path holds the promise of enhancing rehabilitative strategies, ultimately promoting functional recovery and long-term independence for individuals with neuromotor disorders.

## 1 Introduction

Neurological disorders and neurotraumatic injuries often result in severe motor impairments that significantly impact patients' independence (Oczkowski and Barreca, 1993; Catz et al., 1997; Scivoletto et al., 2013) and quality of life (King, 1996; Dijkers, 1997; Westgren and Levi, 1998). However, residual motor activity, which is preserved in many affected patients, can be harnessed to promote neuroplasticity and enhance muscle strength, ultimately resulting in significant functional improvements (Dobkin, 2004; van Hedel and Dietz, 2010; Langhorne et al., 2011; Nas et al., 2015; Stinear et al., 2020; Somers and Bender-Burnett, 2024). For instance, spinal cord injuries (SCI) are typically subdivided into complete and incomplete, with incomplete SCIs sparing at least some sensorimotor functions (Kang et al., 2018). Even in the case of complete SCIs, where no residual sensorimotor function is observable, some studies have suggested that electrical stimulation and intensive rehabilitation may lead to the restoration of voluntary movements (Angeli et al., 2014). Thus, the absence of observable residual function does not necessarily correspond to a complete lack of neural traffic through the cortico-spinal tract, which may be leveraged through rehabilitation (Wahlgren et al., 2021). Similarly, even though stroke typically causes limb paresis contralateral to the produced brain lesion, substantial functional recovery can be attained by exploiting residual motor functions and the plasticity of nearby brain regions (Virani et al., 2020).

When SCI or stroke results in lower limb paralysis, restoring the ability to walk safely and independently becomes a primary goal for affected individuals. In particular, individuals with SCI-related paraplegia consistently rank gait restoration among their highest priorities, second only to the recovery of bladder, bowel, and sexual functions (Simpson et al., 2012). Although stroke more commonly causes unilateral motor impairments, it is estimated that approximately one-third of stroke survivors do not regain independent ambulation (Hendricks et al., 2002). Among those who do, many continue to exhibit pathological gait asymmetries and reduced walking speed (Veerbeek et al., 2014). Furthermore, even in patients who retain some degree of mobility, residual muscle weakness can contribute to balance deficits, a problem exacerbated by advanced age (Beyaert et al., 2015). While regaining the ability to walk is a central aim of rehabilitation in individuals with lower limb paralysis, even achieving upright standing can yield systemic benefits. These include improvements in cardiovascular regulation (Dunn et al., 1998; Eng et al., 2001; Edwards and Layne, 2007), as well as enhanced bowel (Dunn et al., 1998; Walter et al., 1999; Eng et al., 2001; Hoenig et al., 2001; Netz et al., 2007) and urinary function (Dunn et al., 1998; Walter et al., 1999; Eng et al., 2001).

In recent years, a variety of technology-based interventional strategies have been explored as add-ons to traditional physical therapy for the rehabilitation of lower limb function. In this context, here we focus on two key approaches: powered orthoses and exoskeletons, robotic devices designed to provide mechanical support and passive movement to paralyzed limbs (Herr, 2009), and neuromodulation, which facilitates muscle contractions through the electrical stimulation of the neuromuscular system (Hamid and Hayek, 2008; Popović et al., 2009). Initial proof-of-concept studies have demonstrated the potential of these technologies to restore gait (Asselin et al., 2016; Wagner et al., 2018; Haufe et al., 2020; Romeni et al., 2025) and to support the execution of basic functional tasks such as standing (Hankov et al., 2025), sit-to-stand transitions (Li et al., 2023; Romeni et al., 2025), and stair climbing (Hankov et al., 2025; Romeni et al., 2025). These encouraging results have catalyzed efforts to translate such technologies into real-world applications and activities of daily living (van Dijsseldonk et al., 2020; Rowald et al., 2022), which require more sophisticated control strategies as well as improvements in portability and ease of use in unstructured environments.

**Figure 1:**
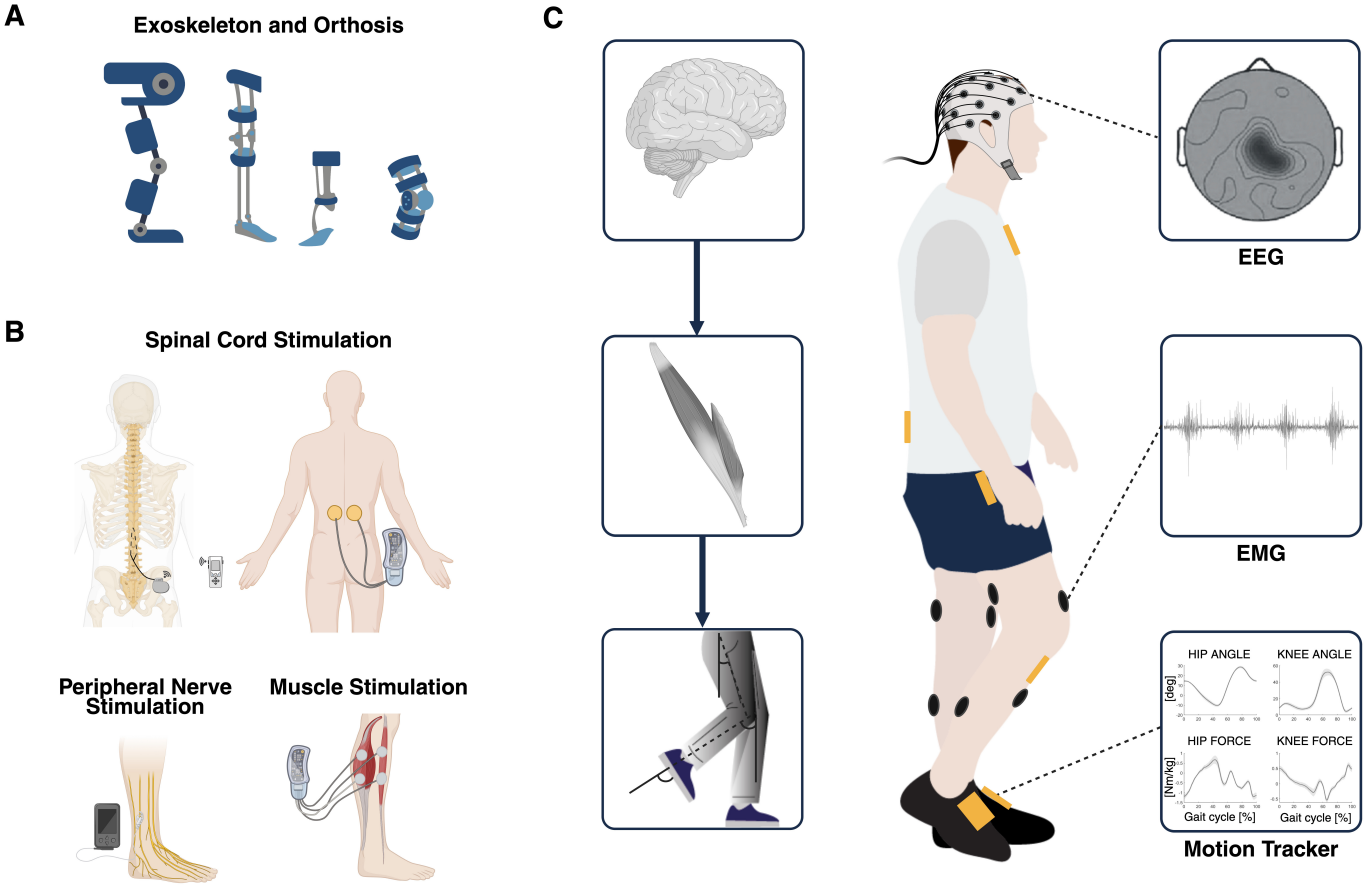
**(A)** Overview of robot-assisted devices such as exoskeletons and powered orthoses used as established interventional strategies for gait recovery. **(B)** Overview of electrical stimulation of the spinal cord, the nerves, and the muscles used as established interventional strategies for gait recovery. **(C)** Multimodal data used to simultaneously explore different domains of the hierarchical organization of the neuromusculoskeletal system: brain, muscles, and biomechanics (MoBI framework). Multimodal biomarkers of residual motor activity can be extracted from EEG, EMG, and kinematics signals and can be exploited to control in real-time different closed-loop interventions aimed at recovering walking through personalized assistance and therapy. MoBI, mobile brain/body imaging; EEG, electroencephalography; EMG, electromyography. Created with BioRender. de Seta V and Romeni S (2025). https://BioRender.com.

Over time, various control strategies have been developed with the dual aim of enabling intuitive and continuous user-driven control to enhance device usability and acceptance (Semprini et al., 2022) and promoting activity-dependent plasticity in the nervous system to maximize neurological recovery (Roy et al., 2012; Mrachacz-Kersting et al., 2019). Wearable mobile brain/body imaging (MoBI) systems, capable of continuously capturing high-density brain and muscle signals along with body movement's kinematics, offer a comprehensive way to optimize and adapt the control of interventional devices (He et al., 2018b) (Figure 1).

In this narrative review, we aim to explore how robotic devices and neuromodulation can be controlled for assistive and rehabilitative interventions, and how different interventional strategies can be integrated to provide personalized gait rehabilitation, leveraging complementary mechanisms of action. First, we will present the state-of-the-art in robotic devices and neuromodulation for lower limb movement restoration; then, we will describe how patients' residual motor activity recorded through various approaches exploiting kinematic, muscle, and neural signals has been used in the past to control such technologies. Finally, we will provide indications and highlight potential issues in the integration of MoBI techniques with different combinations of robotic devices and neuromodulation technologies to achieve functional and physiological recovery of lower limb abilities. The main goal of this review is to provide an overview of rehabilitative interventions for the recovery of lower limb motor functions through the exploitation of residual motor functions, robotic devices, neuromodulation, and monitoring of neurophysiological correlates of movement.

## 2 Interventional strategies for gait recovery

### 2.1 Rehabilitative and assistive strategies

Functional gait restoration can be achieved through a wide range of interventional approaches, broadly categorized as either rehabilitative or assistive. Rehabilitative interventions aim to improve the patient's motor function, promoting the reorganization of the nervous system, with benefits that persist even in the absence of the intervention. In contrast, assistive technologies are designed to support the execution of specific motor tasks, such as walking, without necessarily promoting long-term neuroplastic changes or functional recovery.

Rehabilitative interventions can be further divided into active and passive approaches. Active rehabilitative interventions include all strategies and technologies that enable and promote motor learning/recovery of impaired functions by engaging patients with neurological disorders in functional tasks within a longitudinal training path that leads to generalized motor improvement. Both neuromodulation and exoskeletons might fall into this category. For instance, a gait exoskeleton routinely used for robot-assisted gait training (RAGT), such as the Lokomat Pro (Hocoma, Switzerland) (Jezernik et al., 2003), is used in patients with mild to severe walking impairment to provide therapy by promoting activity-dependent modulation of the proprioceptive pathways under controlled conditions while supporting the patient's body. Indeed, by longitudinally adjusting the level of robotic assistance throughout the rehabilitative path, the patients are challenged to actively contribute to the walking pattern, and this promotes the reorganization of the nervous system, maximizing functional recovery (Riener et al., 2005; Calabrò et al., 2018). Likewise, neuromodulation technologies facilitate the performance of rehabilitative tasks (Romeni et al., 2025), but they also have an impact on synaptic and structural terms (Anderson et al., 2022; Kathe et al., 2022).

On the other hand, passive rehabilitative interventions refer to the ability of electrical stimulation to induce plastic changes in neural pathways, as well as in muscle contractile and structural properties, even when the stimulation is not activity-dependent (Gargiulo et al., 2011; Carraro et al., 2015, 2017). Powered orthoses do not activate paralyzed muscles, but rather mobilize them, and thus they do not exhibit this passive rehabilitation potential.

Whereas, assistive interventions consist of providing constant support during the task by powered orthoses or a stimulation device. Even if the patient improves their performance, the aim is not to get rid of the device, but just to get improved motor performance with the device on (Tyson and Rogerson, 2009; Yan et al., 2015). For example, an active ankle orthosis could deal with foot drop in a purely assistive fashion, thus allowing the foot not to drag during walking, without the need for a targeted rehabilitative effort to remove the orthosis at a later moment.

Rehabilitative and assistive interventions should not be intended as mutually exclusive but should be combined according to the needs and deficits of the patient and the rehabilitative target set by the clinicians, leveraging the complementarity of robotics devices and neuromodulation.

Several maladaptive changes, such as compensatory movements and spasticity, have been described along the process of motor recovery after a stroke or a SCI, addressing them is critical for the effective design and application of both rehabilitative and assistive technologies. Spasticity denotes a set of motor symptoms including increased muscle tone (hypertonia), increased reflexes (hyper-reflexia), involuntary muscle contractions (spasms and ankle clonus), and pathological co-contraction patterns, that have an important prevalence both in stroke (Thibaut et al., 2013) and SCI patients (Sköld et al., 1999; Adams and Hicks, 2005). The integration of robotic devices and neuromodulation should therefore aim not only to restore the motor function but also to promote physiological motor recovery by actively discouraging the emergence or reinforcement of such maladaptive mechanisms.

To better understand the potential of each component to support functional gait recovery and neuromuscular reactivation, the following paragraphs will show the main characteristics of robotic devices and electrical stimulation when used independently.

### 2.2 Lower limb-powered orthoses and exoskeletons

Many powered orthoses have been developed and commercialized to facilitate gait rehabilitation, each providing an environment optimized for the patients' current neurological status (Calabrò et al., 2021; Stampacchia et al., 2022). They are traditionally used to provide body weight support (BWS), assist lower extremity movement compensating, for example, for weak knee extension, and allow repetitive overground walking during conventional physiotherapy (Herr, 2009). According to the patient's level of impairment, they can assist multiple joints (i.e., hip, knee, and ankle) or just a single one (Figure 1A). Both multi-joint (Ortlieb et al., 2017; Laffranchi et al., 2021; Vouga et al., 2022) and single-joint (Wong et al., 2012; Lee et al., 2020) exoskeletons have been developed to assist balance and promote the recovery of physiological gait patterns (Blaya and Herr, 2004). They provide different levels of assistance through their integrated motors that can be personalized according to the patient's needs (Calabrò et al., 2021; Stampacchia et al., 2022). Moreover, the ability of exoskeletons to partially offload the patient's weight may provide BWS during early phases of rehabilitation when more complex systems like the three-dimensional overground BWS system Rysen presented by Mignardot et al. (2017) are not available. Compared to traditional BWS systems, exoskeletons can deliver targeted assistance to key joints, which may be crucial for training independent standing (Emmens et al., 2018). A comprehensive review of lower limb powered orthoses available in 2015 can be found in Yan et al. (2015). Soft exosuits have been recently proposed to address the large masses and rigid design of traditional powered orthoses, especially in unconstrained environments, reducing the metabolic cost of walking in stroke patients (Awad et al., 2017, 2020). For example, Myosuit (MyoSwiss AG, Zurich, Switzerland) is a lightweight, soft exoskeleton that actively supports weight bearing via a tendon cable assisting knee and hip extension (Schmidt et al., 2017). It was shown that it enables individuals with different movement disorders to walk faster with its assistance (Haufe et al., 2019, 2020).

Another compelling feature of powered orthoses is their ability to provide measurements of kinematic and kinetic parameters, such as joint angles, torques, step length, and temporal characteristics of gait phases, through embedded sensors. These measurements offer valuable quantitative insights into patients' motor performance, allowing for objective assessment of their progress throughout the rehabilitation process (Moeller et al., 2023). Moreover, the set of data available in real-time can be employed in a closed-loop to control the orthosis and adapt dynamically the assistance provided during gait (Baud et al., 2021). Finally, passive orthoses can be used in some cases as assistive devices to improve, for example, the foot drop or facilitate sit-to-stand transitions (Berkelman et al., 2007; Eguchi et al., 2018).

RAGT, while useful for sustaining patients during walking and promoting activity-dependent modulation of proprioceptive pathways, has significant limitations that must be addressed to optimize its rehabilitative potential. Substantial changes in muscle coordination patterns have been observed during exoskeleton-assisted walking, not only in patients but also in neurologically intact individuals (Moreno et al., 2013; Sylos-Labini et al., 2014). These alterations may reflect maladaptive changes, such as abnormal spatiotemporal integration of activity in specific spinal segments, which can impede gait recovery or even lead to gait abnormalities (Ivanenko et al., 2009).

### 2.3 Neuromodulation for lower limb movement restoration

Electrical stimulation acts directly on the neuromuscular system and induces muscle contraction by injecting electrical activity into the nervous system through implanted or transcutaneous electrodes (Figure 1B). In order to produce functional muscle contraction, the stimulation target and the downstream neural structures must be preserved, making different stimulation approaches optimal in different clinical scenarios.

Functional electrical stimulation (FES) is a non-invasive technique that targets muscles and neuromuscular junctions and has been investigated for over 50 years (Vodovnik et al., 1967; Ferrarin et al., 2001; Peckham and Knutson, 2005). FES can target single muscle groups to provide targeted rehabilitation or assistance, intervening on the weakness of isolated muscle groups, for example, FES reduced foot-drop and ankle plantar flexor spasticity in stroke patients (Sabut et al., 2011), or to improve standing in paraplegic patients (Hunt et al., 2001). From the early years of FES development, it became clear that gait restoration required multichannel FES systems to target the key involved muscles (Stanic et al., 1978). In Yan et al. (2005), a randomized placebo-controlled trial showed improved outcomes in 46 stroke patients when FES of tibialis anterior, quadriceps, gastrocnemius, and hamstrings muscles were activated in correspondence to the different gait phases, with respect to sham FES and no FES. An attempt to reduce the hardware complexity of FES montages has been based on the observations that superficial stimulation of the peroneal nerve allowed the elicitation of a triple flexion reflex response useful to promote swing (Stefancic et al., 1986; Bajd et al., 1999). In this way, only electrodes on the knee extensors for weight acceptance and on the peroneal nerve for inducing hip flexion can be sufficient to improve gait. A different approach employs implanted FES (iFES), reducing the cluttering of external hardware components by implanting electrodes in the target muscles and allowing for improved walking with walker and crutches, and in a few subjects, allowing stair climbing (Marsolais and Kobetic, 1987). In a study on six incomplete SCI patients, iFES training led to an improvement in gait parameters and a reduction in quadriceps spastic hypertonia (Granat et al., 1993). Standing with single-hand support was attained in SCI patients (Ho et al., 2014), and even sit-to-stand transitions (Triolo et al., 2012).

Even though it is simpler to employ in unstructured environments, iFES requires implanting stimulating electrodes in many key muscles, raising possible concerns about the invasiveness of this technological solution. Moreover, FES can lead rapidly to muscle fatigue (Vromans and Faghri, 2018). Alternatively, it is possible to target multiple muscles with a single electrode array implanted at the level of peripheral nerves, or potentially all lower limb muscles when targeting the spinal cord. Peripheral nerves can be stimulated through cuff electrodes or linear leads to improve standing and dorsiflexion/plantar flexion movements in SCI patients (Fisher et al., 2008; Delianides et al., 2020; Lemos et al., 2023). Transcutaneous spinal cord stimulation (tSCS) targets the spinal cord employing an electric current flux traversing the subject's torso. This technology was introduced in Minassian et al. (2007) and its effectiveness in eliciting motor reflexes in subjects in the supine position has been confirmed in Courtine et al. (2007), Gorodnichev et al. (2012), and Gerasimenko et al. (2015a). It has been tested in complete SCI patients to reinstate passive stepping movements (Gerasimenko et al., 2015b), and it has been shown to improve gait (Hofstoetter et al., 2013, 2020) and spasticity (Hofstoetter et al., 2020, 2021; Minassian et al., 2024). Finally, epidural electrical stimulation (EES) of the spinal cord is an emerging, fully implanted technology that recruits motor fibers primarily through monosynaptic pathways with the targeted afferent fibers in the dorsal roots of the spinal cord (Rattay et al., 2000; Capogrosso et al., 2013). EES has been employed in the past to restore or enhance lower limb functions in patients suffering from complete (Harkema et al., 2011; Angeli et al., 2014, 2018; Rowald et al., 2022) and incomplete (Wagner et al., 2018; Romeni et al., 2025) spinal cord lesions at the thoracic level. While first studies were limited to restoring standing and walking in complete SCI patients (Harkema et al., 2011), even claiming to be able to reinstate voluntary contractions despite the severity of the lesion (Angeli et al., 2014, 2018), later accounts focused more on restoring a wide range of functional activations to enable different motor tasks, including recreational activities (Rowald et al., 2022). In incomplete SCI, important longitudinal improvements have been attained in terms of voluntary muscle strength, walking distances, and walking speed (Wagner et al., 2018). Recently, high frequency EES has shown the capability to suppress spastic hyper-reflexia, hypertonia and to mitigate pathological co-contraction patterns during functional tasks (Romeni et al., 2025).

## 3 MoBI to control interventional strategies

### 3.1 Control strategies for interventional devices

For assistive devices to be effectively used in daily life activities, they must offer an intuitive control interface. Similarly, during active rehabilitation, it has been shown that synchronizing rehabilitative devices with the patient's voluntary movement intention can enhance the reorganization of the nervous system (Jackson and Zimmermann, 2012; Mrachacz-Kersting et al., 2012).

There are various modalities of controlling interventional devices: (i) manual motor state switching through device controllers; (ii) automatic motor state switching, which can be used to control the device based on patient's movement intention and to switch programs based on patient's activity (e.g., sit-to-stand transition, walking, climbing stairs); (iii) adaptive control, where the electrical stimulation parameters and, the assistance and trajectory of the exoskeletons are continuously adjusted to produce the most physiological muscle activation pattern and kinematics. For the automatic motor state switching, a high-level decoding, such as motor intention decoding, is needed, while the adaptive control of the interventional strategy requires low-level control of muscle activation and kinematics (Baud et al., 2021).

When active participation is required from the patient, the ideal solution to control interventional devices would be a so-called “neural bypass,” in which natural motor commands are extracted from brain activity and used to control the device. This is mainly used as high-level control, as it encodes the patient's movement intention rather than precise movement kinematic/kinetic features. A prominent example in the field of spinal cord stimulation is the development of a “brain-spine” interface, which decodes motor intentions from brain activity to trigger electrical stimulation of the nervous system below the injury site (Capogrosso et al., 2016). Recently, it has been demonstrated that an electrocorticography (ECoG)-based brain-spine interface can enable a patient with chronic spinal cord injury to stand and walk naturally (Lorach et al., 2022, 2023). Non-invasive brain signals such as electroencephalographic (EEG) signals have been employed in the past years to control powered orthoses and FES (Shokur et al., 2018; Tariq et al., 2018; Selfslagh et al., 2019) in the context of brain-computer interfaces (BCIs). BCI technologies provide a window into brain mechanisms, directly decoding the brain activity of the users and providing a feedback aligned with the intended action (Wolpaw and Wolpaw, 2012). In stroke patients, BCIs help to reinforce the activity of perilesional areas, enabling them to compensate for lost functions (Soekadar et al., 2015). Importantly, the brain-driven activation of impaired muscles generates sensory feedback that is both temporally and functionally aligned with the intended movement. This congruent feedback is sent back to the brain and enhances motor relearning by promoting neuroplasticity in individuals with incomplete SCI, and drives functional reorganization of the brain after a stroke (Chaudhary et al., 2016). However, EEG suffers from some structural limitations: low signal-to-noise ratio and spatial resolution, susceptibility to environmental and movement artifacts (Vaid et al., 2015). To address these challenges without requiring patients with moderate impairments to undergo surgery for an invasive brain implant, it may be necessary to integrate brain signals with other motor-related signals. The muscle activity of the legs recorded via electromyography (EMG) (Jiang et al., 2010) and lower limb kinematics through wearable motion capture technologies such as inertial measurement unit (IMU) sensors, foot switches, and force sensors can be used to leverage the overall patients' residual motor functions.

**Figure 2:**
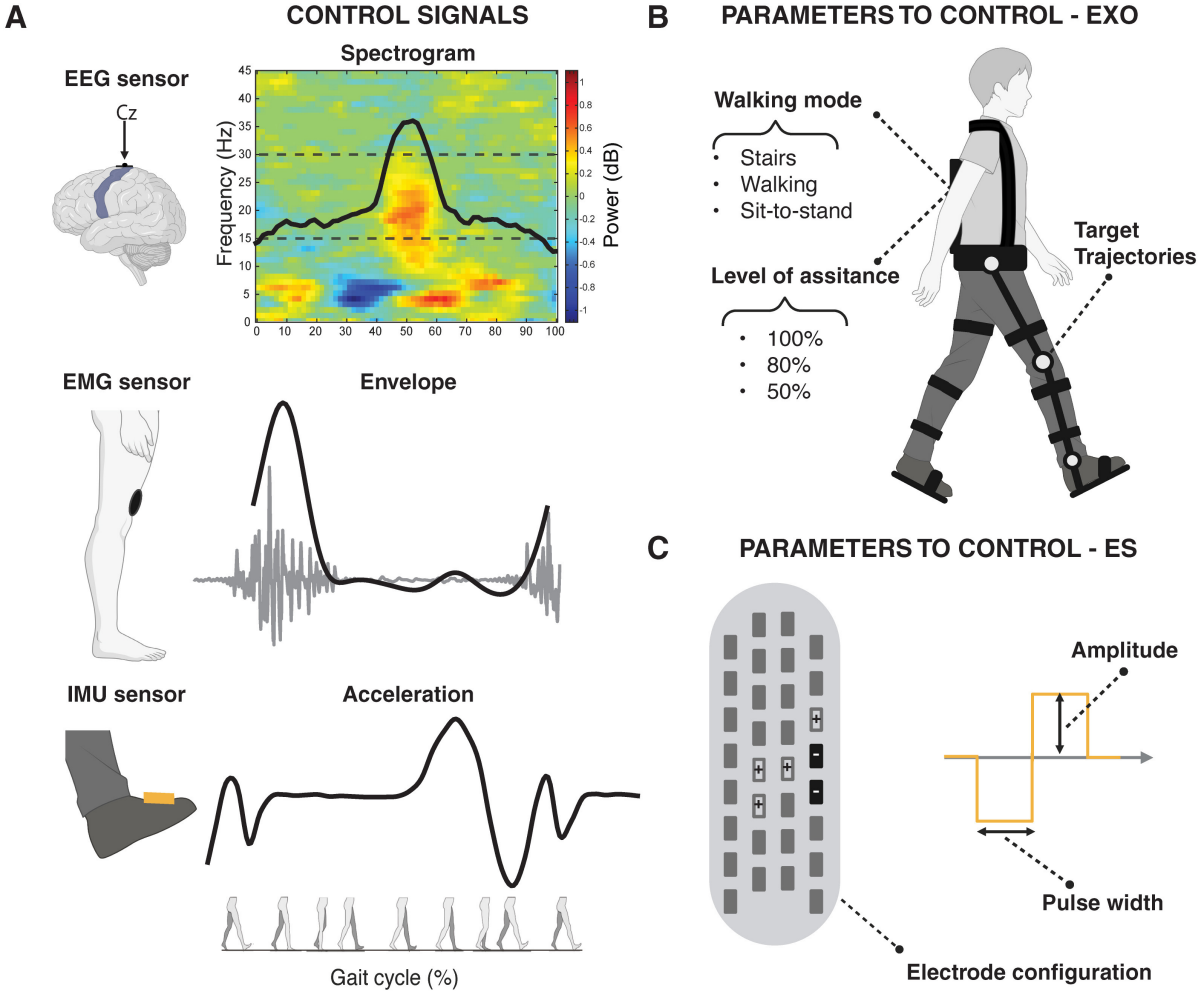
Conceptual representation of distinct gait-related modulations in brain, muscle, and kinematic activity, which can be leveraged as physiological signals to control (i.e., activate or deactivate) and adjust (i.e., tune parameter values of) interventional devices. **(A)** Modulation during a gait cycle of **(Top)** Brain activity can be captured via EEG over the sensorimotor cortex by the time-frequency spectral power and the related modulation curve in the beta band (15–30 Hz); (Center) Muscle activity can be captured through an EMG sensor over a knee extensor muscle by the EMG envelope; **(Bottom)** Kinematic data can be captured via an IMU sensor placed on the foot by the acceleration profile. **(B)** Examples of configurable parameters in robotic devices, such as exoskeletons and powered orthoses (EXO). These include the walking mode (e.g., stairs, speed of walking, sit-to-stand), the level of assistance (e.g., 100%, 80%, 50%), and the target joint trajectories. **(C)** Examples of configurable parameters in electrical stimulation (ES) technologies such as electrode configuration, stimulation amplitude, and pulse width. The figure illustrates a lead used in epidural electrical stimulation (EES) as an example, but the same parameters (with a reduced number of electrodes) apply to other ES techniques, including Functional Electrical Stimulation (FES) and transcutaneous Spinal Cord Stimulation (tSCS). EEG, electroencephalography; EMG, electromyography; IMU, inertial measurement unit.

Figure 1C shows the multimodal techniques able to record signals from the three domains involved in motor recovery (i.e., brain, muscles, and kinematics). Several EEG, EMG, and kinematic parameters can be computed in real-time and used to control closed-loop interventional devices (Stauffer et al., 2009; Shokur et al., 2018; Tariq et al., 2018; Selfslagh et al., 2019). Several studies have explored different EEG correlates for decoding movement intention (López-Larraz et al., 2015; Liu et al., 2017, 2018; de Seta et al., 2024) and evaluated how to optimize the ability of EEG to non-invasively decode gait patterns (Sburlea et al., 2015; Nakagome et al., 2020; Tortora et al., 2020a). Often, the use of EEG to control interventional devices is used in combination with EMG and kinematics through the use of a hybrid approach (e.g., EEG+EMG/kinematics features) (Tortora et al., 2020b) or hybrid features (e.g., cortico-muscular coherence features) (De Seta et al., 2022) to increase the performance and facilitate the encouragement of only physiological patterns along the rehabilitation process (Sarasola-Sanz et al., 2017). EMG parameters can also be used alone to trigger electrical stimulation, to adapt in real-time the stimulation parameters (i.e., low-level control) proportionally according to the level of activation (reinforcement learning), or complementary to the residual or recovered muscular activity of the patient, reflecting the rehabilitative approach of supporting the patient through the functional recovery (Dutta et al., 2008; Jiang et al., 2010; de Seta, 2023). Whereas, IMU sensors, foot switches, and force sensors can detect gait events, kinematic (angle, speed) and kinetic (torque, force) profiles to control powered orthoses and exoskeletons (Baud et al., 2021; Figure 2).

In the following subsections, we will describe how different combinations of physiological signals can be used to control powered orthoses and neuromodulation and assess patients' level of impairment. Figure 2 shows a conceptual example of brain, muscle, and kinematic modulation during gait that can be exploited in real-time as control signals to control and adjust different parameters of the interventional devices.

### 3.2 Mobile brain/body-controlled powered orthoses and exoskeletons

The automatic control of lower limb-powered orthoses has normally been tackled using kinematics data able to track the different phases of the gait cycles (i.e., stance and swing phase) and/or kinetics data based on the interaction forces between the user and the exoskeleton, recorded through sensors, mainly integrated in the system (Baud et al., 2021).

While neurophysiological signal-based control has not been a primary focus in research, neuromuscular control strategy based on threshold-based algorithms on EMG activity showed improvements in both functional ambulation and muscle synergies in subacute stroke patients, suggesting enhanced neuromotor control (Tan et al., 2020). Moreover, techniques like BCIs offer an intuitive way to control rehabilitative devices by leveraging remaining neural pathways and have the added value of promoting brain plasticity and maintaining high patient engagement (Daly and Wolpaw, 2008; Lebedev and Nicolelis, 2017). To optimize the effectiveness and reliability of BCI-controlled exoskeletons, a hybrid (EEG and EMG) approach can be used to control the lower limb exoskeleton to both compensate the residual or recovered muscle activation and increase decoding performances. The residual or recovered motor activity is often associated with pathological synergies, unwanted contractions, and an increase in spasticity (Beauparlant et al., 2013; Beyaert et al., 2015; Prateek et al., 2018). EEG and EMG can also be combined with different relative weights in the control strategy, depending upon the impairment of the target patient. For example, in Gordleeva et al. (2020), an EEG-based approach is proposed for very impaired patients and individuals subjected to spasticity, with an EMG-based approach entering the picture as the rehabilitation improves residual contraction. However, it has been tested only in healthy subjects operating the exoskeleton under different conditions. Moreover, the combined use of EEG and EMG signals offers a promising strategy to detect involuntary movements, such as spasms, by identifying EMG activity that occurs without corresponding cortical signals in the EEG. This information can be used to enable exoskeletons to actively counteract unwanted movements and improve the quality of assisted motion (Pons, 2010). A multimodal approach can also be used to distinguish between different types of movement-related transitions, for example, to distinguish between the movements of starting, stopping walking and independent movement to and from the centerline of the feet (Li et al., 2024). Moreover, the muscle activity can be used to confirm the decision taken by the brain decoder (Shokur et al., 2018). Such BCIs have proven to have both assistive and rehabilitative effects during gait recovery, showing how neurorehabilitation protocols that actively engage the patients' mental and physical activity while providing the patient assistance can be beneficial to regain motor control. Besides, given that safety concerns are primordial in situations where patients have little to no residual motor control, often exoskeletons require upper body engagement (e.g., crutches for balance). Shared-control strategies incorporating sensors to monitor the state of the exoskeleton have been proposed to enhance safety and mitigate the risk of falls (Vinoj et al., 2019). Brain signals can be included in such frameworks, further improving reliability, for example, including the use of perturbation-evoked potentials to develop fall-prevention mechanisms in exoskeletons (Sujatha Ravindran et al., 2022) or the integration of BCI-controlled devices, which increases the user's engagement and awareness (He et al., 2018a; Ortiz et al., 2020). Brain-controlled exoskeletons act on the affected limb to influence the central nervous system through the afferent pathways (in a bottom-up framework) and, at the same time, exploit the brain modulation to detect volition (in a top-down framework), which leads to neuroplasticity and strengthens the cortico-muscular pathway (Contreras-Vidal et al., 2018; Tariq et al., 2018; Benabid et al., 2019). However, even BCI-controlled exoskeletons face limitations, as they do not inherently become therapeutic devices. As Gharabaghi (2016) notes, for a BCI to be considered therapeutic, it must demonstrate reinforcement learning, progressive modulation of brain dynamics along the rehabilitation path, and a direct link between brain dynamics and behavioral improvements related to therapeutic goals.

The significant variability in study protocols, sample sizes, and outcome measures hinders the standardization necessary to evaluate the clinical efficacy of closed-loop lower limb powered orthoses and exoskeletons in individuals with neuromotor disorders (de Miguel-Fernández et al., 2023). This lack of consistency likely reflects the field's current focus on advancing the technological aspects, such as improving reliability, usability, and feasibility, rather than the clinical ones, given the novelty of these systems.

### 3.3 Mobile brain/body-controlled neuromodulation

Since FES is the older electrical stimulation technique, it is also the one where more control strategies have been thoroughly explored. Early studies primarily relied on simple mechanisms to activate or deactivate stimulation, such as foot switches, which are particularly effective in controlling the activation of stimulation to correct drop foot and are thus still largely employed (Kottink et al., 2007; Lee et al., 2014; Melo et al., 2015). More recent approaches have introduced more sophisticated control strategies based on lower limb kinematics and myoelectric signals. Closed-loop superficial FES systems using kinematic variables to promote standing were investigated in several studies (Matjacic and Bajd, 1998; Holderbaum et al., 2002; Gollee et al., 2004). For instance, Matjačić et al. (2003) used superficial FES to control ankle joint movements in paraplegic patients, monitoring balance through kinematic variables while stabilizing the hip and knee joints with an external sensorized orthosis. Similarly, Hunt et al. (2004) showed how the use of cycling-derived kinematic and dynamic data can be used to control FES targeting the quadriceps, hamstrings, and gluteal muscles [for an early review on myoelectric control, see Jiang et al. (2010)]. The feasibility of controlling FES in closed-loop led to its application in more complex and unstructured scenarios, with the impractical placement of electrodes and the lack of portability of the hardware limiting its application in realistic, clinically relevant environments (Braz et al., 2009). Solutions based on iFES were thus developed, for example, implementing EMG-controlled iFES to assist walking (Dutta et al., 2008) or to modulate walking speed (Lombardo et al., 2015). Moreover, stimulation of peripheral nerves through cuff electrodes offers the advantage of activating multiple muscle groups with a single interface, significantly simplifying the hardware requirements for intramuscular FES systems. Christie et al. (2017) reported stability results for peripheral nerve stimulation using cuff electrodes in a large cohort of patients.

The automatic control of electrical spinal cord stimulation remains relatively underdeveloped, in part because continuous subthreshold stimulation has proven sufficient to enhance muscle contractions in patients with residual motor function, both with EES (Romeni et al., 2025) and tSCS (Minassian et al., 2011, 2012). However, the development of spatiotemporal EES patterns triggered by lower limb kinematics, as demonstrated in Wagner et al. (2018), has yielded clinically significant results.

Brain control has been used mostly in rehabilitative settings to monitor subject engagement and maximize rehabilitation outcomes by driving central nervous system plasticity, thanks to the causal association of the cortical activity with the movement intention (Mrachacz-Kersting et al., 2016, 2019). However, the applications of their integration in assistive walking neuroprostheses remain infrequent. In Lorach et al. (2023), electrocorticography, a more invasive alternative to EEG was used to control EES. Moreover, EEG has been shown to successfully decode key lower limb movements involved in locomotion in a chronic SCI patient with an EES implant (Toni et al., 2024). In Atkinson et al. (2025), it was shown that EEG could be used to control tSCS, but it was tested on healthy subjects for a single movement. In Insausti-Delgado et al. (2022), transcutaneous magnetic spinal cord stimulation was controlled through EEG in healthy participants.

### 3.4 Mobile brain/body imaging for assessing recovery

The large number of alternative rehabilitative pathways made possible by current technologies requires the development of techniques capable of assessing the patient's condition at each stage of the rehabilitation process. These assessments should explore the full hierarchical organization of the neuromusculoskeletal pathway (i.e., brain, muscle, and biomechanics as shown in Figure 1C), given the multifaceted consequences of SCI and stroke. For example, kinematic measures such as gait asymmetry and altered walking speed can inform about compensation strategies or level of impairments, while muscular parameters (e.g., muscle synergies, EMG time- and frequency-domain parameters, inter-muscular coherence) reveal changes in motor strategies and help identify pathological co-contractions and spasticity (Barroso et al., 2017). Besides, brain activity recordings shed light on central nervous system reorganization following brain or spinal cord damage (Green et al., 1999; Cramer et al., 2005; Garro et al., 2021). MoBI systems have proven effective in characterizing physiological gait patterns across various walking conditions (Wagner et al., 2012; Artoni et al., 2017, 2023), monitoring the temporal dynamics of motor recovery (Pierella et al., 2020), and assessing the impact of closed-loop devices such as BCIs on neural activity during walking (He et al., 2018b; Tortora et al., 2023). Such multimodal approach allows to perform a comprehensive, quantitative characterization of dysfunctional and maladaptive patterns commonly observed during recovery (Gennaro and de Bruin, 2018; Brambilla et al., 2021; Pichiorri et al., 2023), facilitating patient stratification and tailoring rehabilitation strategies to meet individual needs at different stages of motor recovery (Coscia et al., 2019). Indeed, personalizing the rehabilitative treatments to address the specific needs and impairment level of each patient remains a crucial factor to optimize rehabilitation outcomes (Mignardot et al., 2017; Semprini et al., 2022; Slade et al., 2024). Thus, a multimodal approach allows a global assessment of patients' neuro-biomechanical state during rehabilitation and can inform the design of the closed-loop interventional strategies.

## 4 Integration of MoBI, robot-assisted technology, and neuromodulation

### 4.1 Complementarity of robotic devices and neuromodulation

Each one of the technologies discussed has structural limitations, making each of them only suitable to restore a subset of a patient's motor functions. Most of the available powered orthoses are characterized by bulky designs that constrain their use in controlled laboratory settings and hinder patient comfort, compromising their applicability in real-world scenarios (He et al., 2017). Moreover, the assistive use of powered orthoses and exoskeletons showed limited efficacy in promoting robust activation of the neuromuscular system in patients with neurological disorders, leading to modest motor recovery (Piira et al., 2019). Electrical stimulation, instead, can produce longitudinal motor improvements through the targeted strengthening of key muscles, which can be leveraged during rehabilitation (Roy et al., 2012; Anderson et al., 2022). On the other hand, it can be challenging to achieve the selectivity required for the restoration of a rich repertoire of lower limb movements. For example, spinal cord stimulation is unable to recruit ankle dorsiflexors without a substantial co-activation of ankle plantar flexors (Wagner et al., 2018; Romeni et al., 2025), very likely due to the fact that these muscles are innervated by the same nerve roots (Hofstoetter et al., 2020), making spatially selective stimulation protocols challenging to identify. However, foot drop can be reliably and straightforwardly managed through purely assistive interventions such as the use of an ankle and foot orthosis (Zhou et al., 2022).

Some examples of combinations of neuromodulation and robotic devices for post-stroke lower limb rehabilitation can already be found in the literature, as confirmed by the recent review by Rikhof and colleagues (Rikhof et al., 2024). Systems combining FES with powered orthoses, also referred to as “hybrid assistive systems,” are quite established for regaining motor activity after stroke or SCI (Popovic et al., 1989; Popovic and Popovic, 2006). RAGT combined with spinal cord electrical stimulation has been proposed as an efficient and physiologically relevant approach for rehabilitating people with SCI, with benefits for multiple functions, such as locomotor as well as autonomic functions (Ivanenko et al., 2023). Non-invasive spinal cord stimulation improved overground walking in an exoskeleton in a complete SCI patient, reducing the level of robotic assistance needed, increasing voluntary control over knee movement, improving cardiovascular parameters, and subjective ratings of provided assistance (Gad et al., 2017). In Hankov et al. (2025), it has recently shown that using a closed-loop activity-dependent stimulation with the support of robotic devices commonly used in rehabilitation, such as Lokomat and Myosuit, led to an increase in the lower extremity muscle strength, allowing patients to perform outdoor activities.

Finally, robotic devices can provide assistance while measuring kinetic and kinematic data that can be employed to control electrical stimulation. For example, orthoses and exoskeletons can facilitate gait initiation and stability while measuring interaction forces between the user and the system to fine-tune in real-time electrical stimulation parameters (Stauffer et al., 2009; Hankov et al., 2025), or they can allow closed-loop control of stimulation parameters to achieve a desired joint trajectory (Jezernik et al., 2003). Table 1 provides an overview of the single interventional strategies for gait recovery with their related control strategies, summarizes their key points and limitations, and highlights the potential of the synergistic combination of MoBI, robotics and neuromodulation for regaining locomotor abilities.

**Table 1:** Overview of the key points and limitations of robot-based technologies and neuromodulation techniques used separately and combined for gait recovery, with their relative control strategies and types.

**Interventional devices**	**Control strategies**	**Types of control**	**Key points**	**Limitations**
Robot-based technologies	• Mainly biomechanical data recorded through IMU sensors, foot switches, and force sensors integrated in the exoskeleton or used as external sensors • Brain signals (non-invasive through EEG or invasive through ECoG) • EEG and EMG signals in a hybrid approach	• Mainly adaptive (low level control) • Automatic motor state switching (high level control)	• Body support and movement assistance • Well-established and personalized technologies evolved in the last decades • Provide assisted-as-needed treatment • Maximize neuroplasticity and engagement when brain-controlled	• Do not fully replicate natural gait patterns • Can lead to changes in muscle coordination • Can be technologically complex • Often require upper body engagement (e.g., crutches for balance)
Neuromodulation techniques	• Kinematics data through IMU sensors and foot switches • Myoelectric signal through EMG • Brain signals (non-invasive through EEG or invasive through ECoG)	• Adaptive (low level control) • Automatic motor state switching (high level control)	• Activate the locomotor network and promote plasticity in spinal neuronal circuits • Induce stepping-like movements • Provide assisted-as-needed treatment • Induce neuroplasticity and reorganization of spinal network specially when brain-controlled	• Lack precise selectivity of specific muscle groups needed to produce functional gait (e.g., foot dorsiflexors) • Not able to provide weight bearing activity
Robot-based technologies + Neuromodulation techniques	• Biomechanical data recorded through sensors integrated in exoskeletons or IMU sensors • EEG and EMG signals in a hybrid approach • Brain signals through EEG	• Adaptive (low level control) of the electrical stimulation parameters • Adaptive (low level control) of exoskeletons' level of assistance and trajectories • Automatic motor state switching (high level control)	Synergistic combination of the key points of the single technologies: • Body support, continuous kinematics monitoring and necessary locomotor training provided by less rigid exoskeletons • Direct action on the cortico-muscular network (“ecological gait”), reinforcement of physiological gait patterns, stimulation-induced neuroplasticity provided by neuromodulation techniques	• Need to understand long-term effects • Technical requirements for real-time synchronization of different systems during locomotion

IMU, inertial measurement unit; EEG, electroencephalography; ECoG, electrocorticography; EMG, electromyography.

The set of robotic and neuromodulation technologies employed on the same patient may change over time and can combine the rehabilitative and assistive aspects of these technologies (Hankov et al., 2025). For example, passive electrostimulation could be employed to strengthen muscles before the start of the rehabilitation, so that the residual neural drive available may be enough to produce sufficient strength. As another example, the rehabilitative path of a patient could benefit from assistive technologies to focus on rehabilitating a target function without worrying about other deficits. For instance, an ankle orthosis for foot drop could be employed when training the hip flexion for ambulation, so that the patient may focus on the rehabilitation of a single movement. When good motor outcomes have been obtained with respect to the movement target of the rehabilitation, it can be decided that it is worth/possible to undergo rehabilitation of the movement that has been passively assisted through the orthosis, Table 2.

**Table 2:** Dynamic integration of robotic devices and neuromodulation techniques across rehabilitation stages.

**Rehabilitation stage**	**Integration of robotic devices and neuromodulation techniques**
Early stage	Robotic devices provide critical mechanical support (movement, balance, BWS) to allow training.
Mid stage	Synergistic work of robotics devices and electrical stimulation: robots move limbs, sensors detect movement and trigger precise electrical stimulation.
Late stage and daily living	Gradually reduce robotic assistance as voluntary control improves (assisted-as-needed approach); shift to lighter support devices (e.g., Myosuit).

BWS, body weight support.

### 4.2 Toward a multimodal framework for personalized gait recovery

Recent technological advancements have led to the definition of a large set of technology modules that can be combined according to the patient's needs, as described in Shokur et al. (2021). In such work, the idea of modularity has been introduced with the main purpose of reusing the same technology modules across different neuroprosthetic devices: for instance, Utah array electrodes to decode motor intentions from the brain (Collinger et al., 2013) or to produce tactile sensory feedback through intracortical electrical stimulation (Flesher et al., 2016), or again to be implanted into peripheral nerves of arm amputees to concurrently record movement intention decoding and stimulate sensory feedback (Wendelken et al., 2017). In the current state of rehabilitative and assistive technologies, personalization is a crucial step (Borton et al., 2013) and such modularity approach can be used to address personalization in gait recovery due to the large functional and anatomical variability between patients even inside the same condition, i.e., lesion characteristics (lesion type, area, etc.) and individual characteristics (age, gender, comorbidities, etc.). Recognizing that no single technology can address all patient needs has led to the development of multimodal frameworks where each neurotechnology module can be supported by multiple technological solutions (del-Ama et al., 2014). This modularity principle is at the core of MoBI, and allows to exploit the whole hierarchical organization of the neuromusculoskeletal system to extract more efficiently relevant biomarkers of a patient's level of impairment and exploits such information as motor commands to control and adapt the interventional device. It also inspires the creation of hybrid neuroprosthetic approaches, where multiple interventional strategies can coexist and be adapted based on the quantitative patient profile provided by MoBI to address their needs. This synergistic integration allows for personalized therapy, maximizing functional recovery and providing the patient with an intervention that resembles as much as possible the physiology of movement. Indeed, walking is a dynamic process requiring continuous integration of motor commands and sensory feedback. To replicate physiological gait control in individuals with motor impairments, an effective strategy should involve the combination of high-level (i.e., automatic motor switching) and low-level (i.e., adaptive control) control mimicking what happens during physiological movement. In healthy individuals, the brain initiates movement intentions, while spinal circuits and muscle synergies handle detailed execution without conscious effort. Recreating this hierarchical control structure in rehabilitation devices enhances both naturalness and efficacy. Therefore, high-level decoding should rely on brain signals such as EEG to capture movement intentions and drive motor state transitions (e.g., gait initiating walking). These intentions occur at relatively slow temporal scales and can trigger specific motor programs (He et al., 2018a). While low-level adaptation should rely on signals from EMG and wearable motion sensors, reflecting the spinal and muscular execution of movement (Boonstra et al., 2015; Zipser-Mohammadzada et al., 2023), and ensure physiological coordination, adjust device assistance dynamically, and counteract pathological movement patterns, all without requiring direct patient control. This dual-level framework enables closed-loop devices to simultaneously promote neuroplasticity, personalize support according to residual abilities, and facilitate challenging yet feasible rehabilitation.

Indeed, brain control has demonstrated its efficiency in controlling, in a natural way, assistive devices for gait while promoting neuroplasticity for motor recovery (Belda-Lois et al., 2011). When combined in a multimodal approach with additional physiological measures, such as monitoring functional and dysfunctional muscle patterns or comparing reference kinematics with actual joint angles, it transforms into a personalized and optimized rehabilitation tool (Selfslagh et al., 2019).

**Figure 3:**
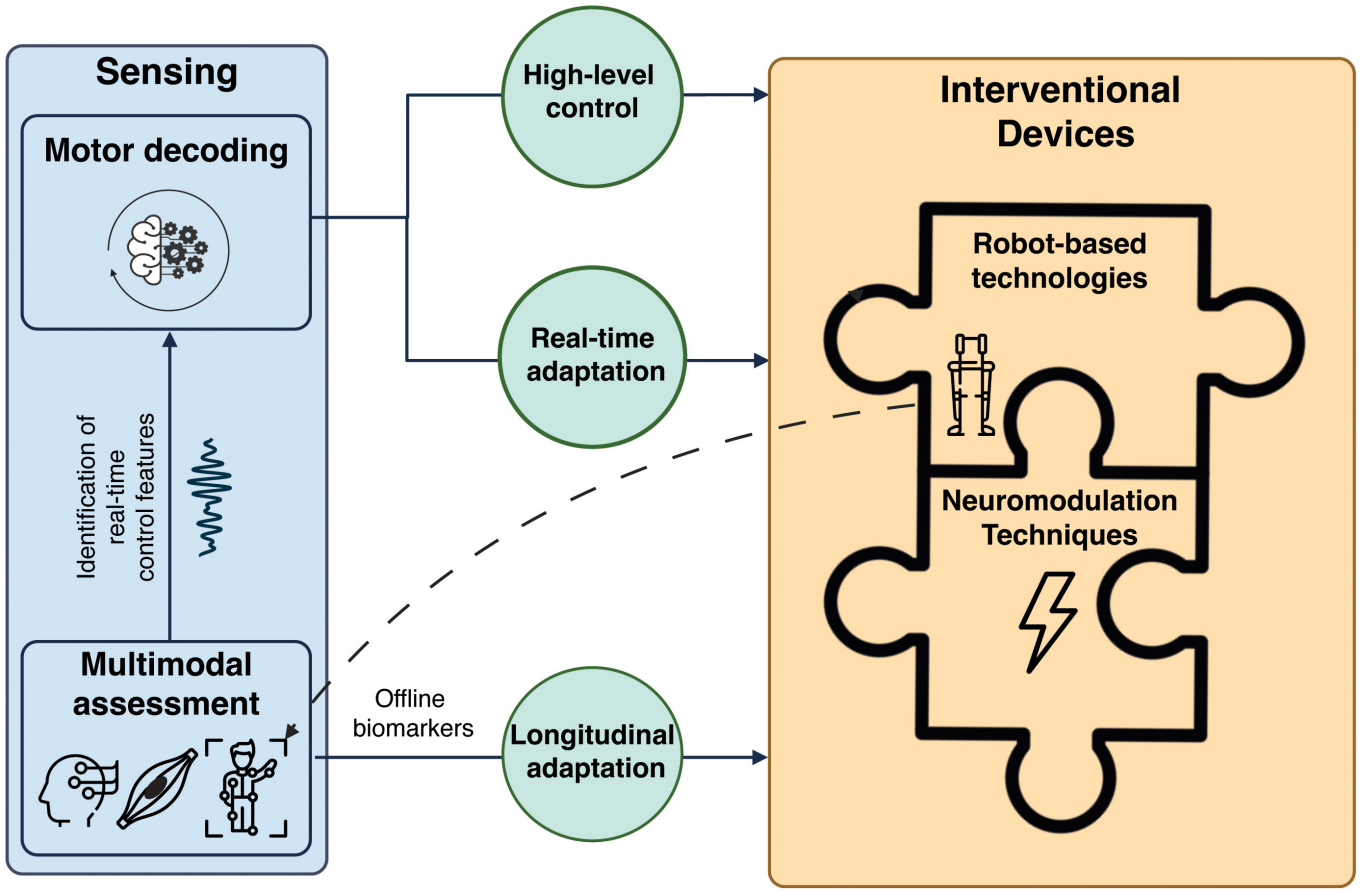
Multimodal framework for motor recovery based on the synergistic integration of mobile brain/body imaging (MoBI), robot-based technologies, and neuromodulation techniques. Multimodal data (i.e., brain, muscle, and kinematics signals) are collected during assessment sessions and used to identify real-time control features to use for the high-level control of the interventional technologies (automatic motor state switching) and the online adaptation of the interventional parameters (i.e., stimulation parameters, exoskeletons' levels of assistance and trajectories) based on patient's intention and residual motor activity. Exoskeletons and powered orthoses, thanks to their integrated sensors, can also be used to record kinematics data. Robot-assisted technologies and neuromodulation devices can be used in a complementary fashion as different components of an integrated interventional technology. The contribution of each single technology and its control features can be adjusted along the rehabilitation path (longitudinal adaptation) based on offline biomarkers of patient's level of impairment, providing personalized rehabilitative treatments.

Finally, regular multimodal assessments along the rehabilitation path allow for dynamic adjustment of control parameters and contribution of each interventional strategy (i.e., robotic devices and neuromodulation) as the patient progresses based on objective and quantitative biomarkers. This supports longitudinal, personalized therapy (Coscia et al., 2019). Figure 3 shows a schematic representation of the multimodal framework for gait recovery based on the synergistic integration of MoBI, robot-based technologies, and neuromodulation techniques. However, the long-term effects of such approach need more research to translate these technologies into viable clinical solutions.

### 4.3 Technical requirements to couple MoBI systems with interventional devices

The development of multimodal closed-loop systems capable of translating from laboratory environments to clinical and home settings presents several important challenges. First, non-invasive sensing methods should be preferred to invasive ones when possible, as they enable recordings over broader areas, providing a global picture of the patient's status and avoiding risks associated with surgical procedures. However, non-invasive technologies exhibit some disadvantages with respect to fully implantable technologies. For example, they are more sensitive to artifacts, even though they provide neuromechanical biomarkers with robustness similar to or higher than conventional clinical scales (Garro et al., 2021). Additionally, despite procedures such as sensor placement and calibration can challenge their adoption, particularly for users with motor impairments or limited caregiver support, several technological advancements have been introduced in the last decade to mitigate this problem. Dry EEG systems (Kleeva et al., 2024) or fully wireless systems (Niso et al., 2023) can facilitate quick and reliable brain data acquisition (Song and Nordin, 2021). Similarly, EMG and inertial sensors must be designed to tolerate minor placement errors and provide consistent data, even when positioned by patients. Medium-density EMG sleeves and armbands improve everyday usability, reducing sensitivity to placement errors even when positioned by patients (Tan et al., 2012; Artoni et al., 2019). Finally, sensors integrated into powered orthoses can be used for data collection in home and community settings for extensive periods of time (Bonato et al., 2024).

Moreover, the reliability of neural signals is often compromised by movement artifacts during ambulatory tasks, which can decrease decoding accuracy and affect user safety (Gorjan et al., 2022). Therefore, real-time artifact mitigation and signal processing are critical for effective deployment in real-world settings, requiring preservation of the optimal trade-off between movement classification accuracy and processing speed (De Seta et al., 2022). A multimodal approach offers a promising strategy to address the limitations of EEG-based control by leveraging additional sources of residual motor activity to improve decoding robustness. Furthermore, employing brain signals primarily for high-level control, such as triggering sequences of motor commands mimicking the physiology of movement, reduces the need for rapid, continuous signal decoding. Instead of requiring the detection of fast dynamics, such as single gait phases, this strategy enables the use of more complex processing techniques than simple filters to remove physiological artifacts, such as moving averages, thresholding of meaningful parameters (e.g., channel variance) and wavelet transform, which can improve signal reliability and reduce sensitivity to transient artifacts (Tariq et al., 2018).

Finally, real-time processing with minimal latency is often required, for example, to prevent falls or unsafe movements during overground walking (Vinoj et al., 2019), particularly during daily life activities, where patients engage different terrains and can be perturbed through interactions with the surrounding environment. Closed-loop systems require being time-locked to motor intentions and efforts, and thus, a precise time synchronization across multiple devices should be granted to avoid issues like jitter and latency, ensuring reliable solutions (Artoni et al., 2017; Iwama et al., 2024). Finally, integrated interventional devices should require minimal recalibration across different sessions, with dedicated optimization sessions in clinical settings ensuring their effective and independent use in real-world scenarios (Hankov et al., 2025). These considerations highlight broader challenges related to real-world usability, underscoring the need for future research to focus on overcoming technical, practical, and usability challenges. Addressing them will be essential to advance multimodal closed-loop systems toward becoming viable clinical and everyday solutions for gait rehabilitation and assistance.

Implementing such a multimodal approach requires the presence of multidisciplinary teams within rehabilitation clinics, comprising clinicians, engineers, and physiotherapists to ensure effective integration and individualized patient care. Moreover, there is a need for technological platforms that are easy to use by non-expert users, such as clinicians and patients, and that can seamlessly adapt and integrate different interventional technology modules.

## 5 Conclusions

The physiological recovery of lower limb functions is a priority after events such as a stroke or a SCI. Multimodal approaches integrating in a synergistic way different interventional and control strategies have the potential to address the challenges related to the reinstatement of complex motor behaviors. While some studies have already explored this integration, with this narrative review, we aim to contribute to the outline of a practical framework for designing personalized rehabilitative treatments that leverage patients' residual motor activity. Advances in mobile brain/body imaging, electrical stimulation, and powered orthoses open promising avenues for modular, cost-effective neuroprostheses that adapt to the patient's specific needs. These flexible systems can decode motor intention, monitor residual motor activity, and address pathological movement patterns in real-time, promoting neuroplasticity and facilitating more natural and efficient recovery processes.

With this approach, patients can be supported from the early stages of their motor disorders through an integrated technology that delivers intensive and safe rehabilitation. This is achieved by combining body support, continuous kinematic monitoring, and essential locomotor training through less rigid exoskeletons, together with direct modulation of the cortico-muscular network and reinforcement of physiological gait patterns via stimulation-induced neuroplasticity provided by neuromodulation techniques. The modularity of this strategy enables continuous optimization and personalization of the intervention according to the patient's evolving functional motor needs across multiple clinical optimization sessions.

Thus, future efforts in gait recovery after neuromotor disorders should prioritize exploiting residual motor functions where available while leveraging neuromodulation and robotics to induce physiological muscle contractions, even in cases of complete paralysis. By reinforcing movement patterns that closely resemble physiological activation, these interventions promote structural and functional reorganization of the nervous and muscular systems. Ultimately, well-powered clinical trials are needed to evaluate the therapeutic potential of this multimodal framework and to allow its clinical translation. The development of synergistic and personalized recovery systems can transform rehabilitation, enabling patients to achieve optimized outcomes through strategies that complement one another and adapt to their individual needs. These systems hold the potential to redefine the boundaries between rehabilitation and assistance, empowering patients with a pathway toward regaining independence and mobility.
